# Exploring Diverse Bioactive Secondary Metabolites from Marine Microorganisms Using Co-Culture Strategy

**DOI:** 10.3390/molecules28176371

**Published:** 2023-08-31

**Authors:** Xiaolin Li, Huayan Xu, Yuyue Li, Shengrong Liao, Yonghong Liu

**Affiliations:** 1 Research Center for Marine Microbes, CAS Key Laboratory of Tropical Marine Bio-Resources and Ecology, Guangdong Key Laboratory of Marine Materia Medica, South China Sea Institute of Oceanology, Chinese Academy of Sciences, Guangzhou 510301, China; 2 University of Chinese Academy of Sciences, Beijing 100049, China; 3 Wuya College of Innovation, Shenyang Pharmaceutical University, Shenyang 110016, China

**Keywords:** marine-derived microorganism, mono-culture, co-culture, secondary metabolites, structural diversity, bioactivity

## Abstract

The isolation and identification of an increasing number of secondary metabolites featuring unique skeletons and possessing diverse bioactivities sourced from marine microorganisms have garnered the interest of numerous natural product chemists. There has been a growing emphasis on how to cultivate microorganisms to enhance the chemical diversity of metabolites and avoid the rediscovery of known ones. Given the significance of secondary metabolites as a means of communication among microorganisms, microbial co-culture has been introduced. By mimicking the growth patterns of microbial communities in their natural habitats, the co-culture strategy is anticipated to stimulate biosynthetic gene clusters that remain dormant under traditional laboratory culture conditions, thereby inducing the production of novel secondary metabolites. Different from previous reviews mainly focusing on fermentation conditions or metabolite diversities from marine-derived co-paired strains, this review covers the marine-derived co-culture microorganisms from 2012 to 2022, and turns to a particular discussion highlighting the selection of co-paired strains for marine-derived microorganisms, especially the fermentation methods for their co-cultural apparatus, and the screening approaches for the convenient and rapid detection of novel metabolites, as these are important in the co-culture. Finally, the structural and bioactivity diversities of molecules are also discussed. The challenges and prospects of co-culture are discussed on behave of the views of the authors.

## 1. Introduction

In the past century, microbial natural products have been a primary source of pharmaceuticals attributing to their structural diversities and fruitful bioactivities [1]. For example, in 1928, British microbiologist Alexander Fleming accidentally discovered penicillin from microorganisms, and thereafter, the use of penicillin in controlling bacterial infections has made great success. Encouraged by these positive results and the demand for the treatment of other diseases, the discovery of antibiotics has entered a golden age, and peaked in the mid-1950s [2]. However, one of the greatest medical dilemmas of the 20th century is the presence of bacterial resistance to antibiotics which are rapidly becoming ineffective in clinical practice, a sharp and seemly unsolvable problem most probably originated from the broad judicious and injudicious use of antibiotics, as well as the incorrect disposal of their waste [3]. In order to solve this problem, several measures have been taken at different levels. These measures are listed as follows: (1) discovering new antibiotics; (2) exploring new biological targets in microbial cells; (3) chemical modification of the existing antibiotics; (4) rationally controlling the use of antibiotics and carefully treating their wastes; (5) developing vaccines. Among all these measures, the discovery of novel bioactive antibiotics by using modern techniques to avoid the rediscovery of secondary metabolites from microbes are promising to alleviate this problem [4]. Indeed, natural products coming from plants, animals, or microorganisms, and synthetic compounds designed based on natural scaffold play an important role in new drug discovery. For instance, from the 1970s to 2019, one-third of the approved drug breakthroughs were related to natural products, including unaltered natural product (3.8%), natural “Botanical” ones (0.8%), and their semi-synthetic derivatives or analogues (18.9%). In addition, there are many delicate total synthetic drugs whose pharmacophores were also inspired by natural products [5]. However, the less availability of novel bioactive secondary metabolites and the increasing rate at which known compounds are being rediscovered make this approach increasingly disappointing. Delightedly, on the basis of natural products, the utilization of genomics, synthetic biology, chemical informatics, bioinformatics, or synthetic chemistry has significantly advanced the discovery of drug candidates, which were widely used in a diverse range of human diseases [5,6].

The activation of ubiquitous orphan biosynthetic gene clusters existed in microbial genome sequences has increasingly attracted researchers’ interests by employing modern biological or chemical techniques, such as genomics, transcriptomics, proteomics, and metabolomics. The silent biosynthetic gene clusters in selected organisms can be activated to induce chemical diversity that would not occur under normal laboratory culture conditions. The genome sequence can be artificially altered via approaches such as gene knockout, promoter exchange, and transcription factor overexpression combined with heterologous or homologous expression [7,8]. In addition, ultraviolet (UV) mutagenesis and metagenomics have been used to remold the microbial genome or activate its silent gene clusters aiming to induce novel metabolites [9]. Alternatively, epigenetic protein modifiers or inhibitors are added to regulate the structure of chromatin, thereby affecting the transcription of different gene clusters, which resulted in the alteration of the biosynthesis of the metabolites [10]. Adding enzyme inhibitors and non-natural substrate to the culture are common ways to regulate microbial biosynthesis. When enzyme inhibitors are added to the medium, some biosynthetic pathways may be blocked, while other silent or poorly expressed gene clusters may be activated or enhanced. Feeding the microorganisms with unnatural substrates is an attempt to guide the synthesis of more active and drug-related compounds, when the corresponding biosynthetic pathway of the target compound is known [11,12]. One strain-many compound (OSMAC) strategy has been shown to be a promising tool for activating silent biological gene clusters. In this strategy, changing the pH value and temperature, controlling the supply of oxygen, carbon, nitrogen, and/or phosphorus sources, and adding metal ions or inducers (such as the enzyme inhibitors described above) can create complicated living environments for microorganisms [13].

Unlike the above-mentioned approaches, microbial co-culture is a strategy which mimics the natural living environment full of competition and communication between microorganisms. Co-cultured microorganisms gain a competitive advantage over mono-culture by up-regulating or down-regulating their corresponding gene clusters, leading to the production of new types of metabolites [14,15]. In 2014, Bertrand and his partners classified and summarized several methods that had been developed to activate silent biosynthetic pathways of metabolites. They highlighted multiple methods for conducting co-culture experiments, discussed analytical methods on studying these interactions, and summarized the chemical diversity and biological activity of the induced metabolites [16]. In 2018, Nai and co-workers discussed the applicability of co-culture with other tools including microfluidic technology, next-generation 3D bioprinting, and single-cell metabolomics [17]. In 2019, Tomm and co-workers discussed several microbial culture methods which included the addition of physical scaffolds, small-molecule activators, and co-culture with another microbe [18]. In 2020, the role of secondary metabolites involved in co-culture systems as a “chemical language” in microbial communication was summarized. These interactions include chemical attack or defense, anti-phage infection, predation and anti-hunting, resource competition, chromosomal remodeling, and chemical space expansion [19]. In 2021, Boruta et al. compared and analyzed the effects of co-culture strategies on the biosynthesis ability of *Streptomyces* from the aspects of the design of co-culture experiments, experimental devices, and factors affecting the results of co-culture [20]. In 2023, Xu summarized new secondary metabolites derived from the fungal co-culture extracts [15]. Alanzi et al. reviewed 155 compounds produced by *Aspergillus* co-culture with bacteria or fungi from 2012 to 2022 [21].

Marine-derived microorganisms’ morphological characteristics, survival strategies, metabolic pathways, and metabolites are quite different from terrestrial microorganisms because of their unique living environment, such as high pressure, high salinity, oligotrophic, and limited light [22]. “OSMAC” emphasizes that a single strain produces different secondary metabolites under different growth environments. Romano et al. summarized the application of the “OSMAC” strategy in marine-derived microorganisms. They discussed the effects of culture parameters such as temperature, nutrient regimes, pH value, and inoculation rate on microbial secondary metabolites. Apart from the “OSMAC” strategy, they also explored the differences of secondary metabolites produced by marine-derived microorganisms in co-culture and mono-culture [23]. Caudal et al. reviewed the structural and activity diversity of metabolites from marine-derived co-culture strains. They discussed the selection of culture techniques, including medium components, physical parameters of fermentation, and inoculation when the co-culture strategy was applied to marine-derived microorganisms [24]. Different from previous reviews [25,26] mainly focusing on fermentation conditions or metabolite diversities from marine-derived co-paired strains, this review covers the marine-derived co-culture microorganisms from 2012 to 2022, and turns to a particular discussion highlighting the selection of co-paired strains for marine-derived microorganisms, especially the fermentation methods for their co-cultural apparatus, and the screening approaches for the convenient and rapid detection of novel metabolites, as these are important in the co-culture. Finally, the structural and bioactive diversities of molecules are also discussed. The challenges and prospects of co-culture are discussed.

## 2. How to Pair Strains for Co-Culture?

### 2.1. Based on the Ecological Niches

Niche refers to an area that is occupied by different microorganism populations which communicate with each other to form a special ecosystem under certain temporal and spatial latitude conditions. Microorganisms that live and reproduce in this ecological environment compete and cooperate due to limited nutritional resources.

**Figure 1:**
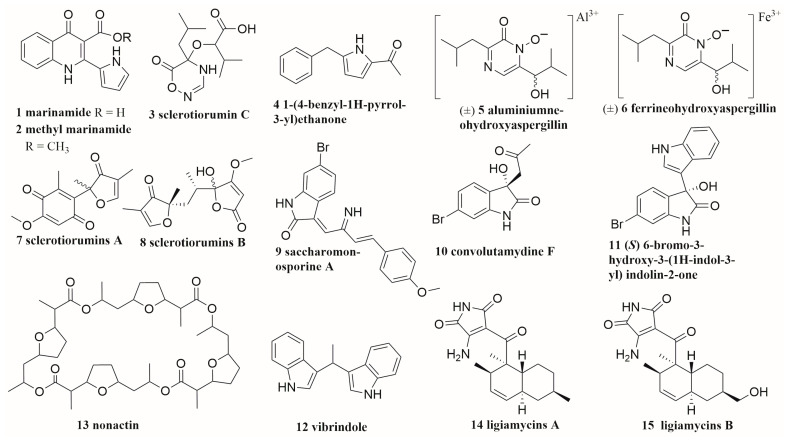
The structures of compounds **1**–**15**.

Marine-derived endophytes are found to exist between or within the cells of marine organisms, including marine plants (algae, seagrass, driftwood, and mangrove), marine vertebrates (mainly fish), and marine invertebrates (mainly sponges and corals) [27]. Zhu et al. co-cultured two unidentified mangrove endophytic fungi (Nos. 1924 and 3893, isolated from the coast of South China Sea) and isolated two new compounds (**1**, **2**) (Figure 1) [28]. Bao et al. isolated six new secondary metabolites (**3**–**6**, **7**, **8**) from the co-culture extracts of marine-derived fungi *Aspergillus sclerotiorum* and *Penicillium citrinum*. The two strains were isolated from gorgonian *Muricella flexuosa* collected from the South China Sea [29]. In 2018, Hawary and his colleagues isolated two novel metabolites, named Saccharomonosporine A (**9**) and convolutamydine F (**10**), and three new compounds (**11**, **12**, **13**) from the co-cultured extract of *Saccharomonospora* sp. UR22 and *Dietzia* sp. UR66 are both derived from the Red Sea sponge *Callyspongia siphonella* [30]. The co-cultivation of marine wharf roach gut bacterial strains *Streptomyces* sp. GET02.ST and *Achromobacter* sp. GET02.AC induced the amount of the new natural product, ligiamycins A (**14**), 24 times more than that only from GET02.ST in mono-culture. It also led to the discovery of a new natural product, ligiamycins B (**15**) (Figure 1) [31]. Although endophytes are considered a promising resource for the discovery of superior co-culture strains, Knowles and his colleagues thought that the endophyte community depends not only on the host but also on the geographical location [32].

In order to survive in special environments such as hydrothermal and cold springs containing extreme pH, temperature, salinity, and pressure, microbial communities have developed unique defenses against their environment, leading to the biosynthesis of new molecules [33]. In anaerobic environments, for example, different metabolic processes are either stimulated or dependent on other partners [4,34]. The complexity of interactions among microbial communities growing in special environments makes them suitable co-culture partners. Examining microbial communities’ laboratory cultivability and interaction in special environments is a possible direction for enriching the natural product library.

### 2.2. Pairing with Pathogens

**Figure 2:**
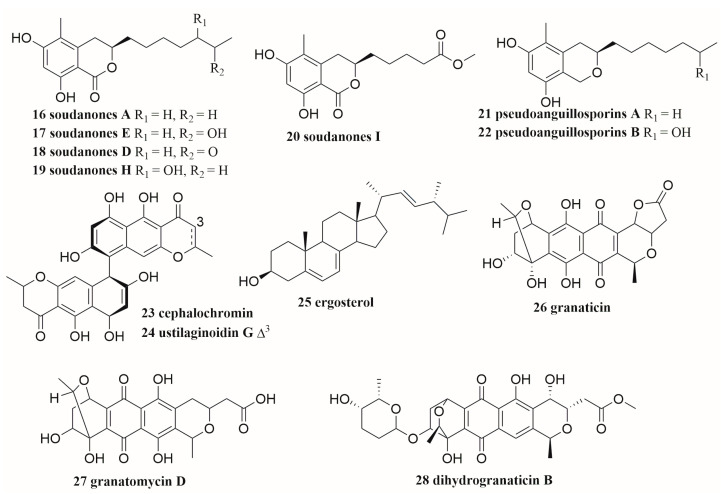
The structures of compounds **16**–**28**.

Globally, plant pathogens threaten a substantial portion of food production, with a 20–30% loss of crops estimated, primarily in food-deficit areas [35]. Danquah et al. obtained 21 fungi via screened from 121 fungi which isolated from the samples of water, sediment, seabed foam, and plant materials (seeds, branches, leaves, sawdust) collected at the Windebyer Noor. They used these genetically diverse fungal strains to co-culture with two phytopathogenic bacteria (*Pseudomonas syringae* and *Ralstonia solanacearum*) and two phytopathogenic fungi (*Magnaporthe oryzae* and *Botrytis cinerea*), respectively, in two pre-selected solid media Sabouraud medium (SA) and Potato Dextrose Agar medium (PDA). The extracts of nine co-cultures showed biological inhibition ≥70% at a concentration of 200 μg·mL^−1^, targeting at least three plant pathogenic test strains [36]. To highlight these results, Danquah et al. chose to conduct a more in-depth study on the co-culture extracts of marine *Cosmospora* sp. and phytopathogen *M. oryzae*. Ten compounds, including two new isochroman-1-one derivatives, soudanones H-I (**19**, **20**) and their three known homologues (**16**, **17**, **18**), and other five known compounds, namely pseudoanguillosporins A and B (**21**, **22**), cephalochromin (**23**), ustilaginoidin G (**24**), and ergosterol (**25**), were identified from the extracts of large-scale co-culture of *C.* sp. and *M. oryzae* (Figure 2) [37]. In addition, they experimented with a new alternative co-culture method, which classified eight marine sediment-derived fungi (*Helotiales* sp., *Plenodomus influorescens*, *Penicillium bialowiezense*, *Sarocladium strictum*, *Pyrenochaeta* sp., *Pyrenochaeta nobilis*, and two *Lentithecium* strains) as “strong” or “weak” categories based on their extraction-resistant abilities to six phytopathogens (*Erwinia amylovora*, *P. syringae*, *R. solanacearum*, *Xanthomonas campestris*, *M. oryzae*, and *Phytophthora infestans*). Then, the co-culture of the three modes was implemented, including pairings of weak–weak, strong–strong, and weak–strong. Finally, the co-culture extracts of *Plenodomus influorescens* (strong), and *Pyrenochaeta nobilis* (weak) showed significant differences in biological activity and metabolic profiles [38].

The presence of resistant pathogens can create a survival pressure on antibiotic-producing strains, forcing antibiotic-producing microorganisms to produce new antibiotics or up-regulate the original antibiotic, thus gaining a competitive advantage [39]. Sung et al. co-cultured the tunicate-associated bacterium *Streptomyces* sp. PTY087I2 with four human bacterial pathogens (*Bacillus subtilis*, methicillin-sensitive *Staphylococcus aureus*, methicillin-resistant *Staphylococcus aureus* (MRSA), and *Pseudomonas aeruginosa*). The molecular network of the co-culture extracts of *S.* sp. with these human pathogens showed that the increased production of three antibiotics including granaticin (**26**), granatomycin D (**27**), and dihydrogranaticin B (**28**) (Figure 2), and several known analogues were discovered. The biological activity of co-cultured strain extracts against Gram-positive human pathogens used in these experiments was significantly increased [40]. Three types of strains, namely heat-killed bacterial pathogens, living bacterial pathogens (*S. aureus, B. subtilis, E. coli*, and *P. aeruginosa*), and environmental strains (*Streptomyces* and *Arthrobacter*), were used to co-culture with *Streptomyces* sp. AC117. The result showed that adding living pathogenic bacteria or phylogenetically closed bacteria to AC117 did not significantly improve the antibacterial activity, while co-cultured with heat-killed bacteria (especially for *S. aureus*), the antibacterial activity of the extracts from AC117 was enhanced, as well as a five-fold increase for the yield of anthraquinones [41]. Ozkaya et al. co-cultured five marine sponge-derived fungi, namely *Cladosporium* sp. (SF17), *Penicillium Commune* (SF42), *Aspergillus carneus* (SF114B), *Penicillium canescens* (SF146A), and *Aspergillus iizukae* (SF153A), with the mixture of four fish pathogenic bacteria *Vibrio anguillarum* O1, *Vagococcus salmoninarum*, *Yersinia ruckeri*, and *Lactococcus garvieae*, respectively. The co-culture induced the increase in antibacterial activity of the two strains: *A. iizukae* (SF153A) and *A. carneus* (SF114B). The mixed fermentation extract of *A. iizukae* showed strong inhibitory activity against *L. garvieae*, with a minimal inhibitory concentration (MIC) value of 1.6 μg·mL^−1^ [42].

### 2.3. Pairing with Sub-Inhibitory Concentration Antibiotics

Some work suggested that secondary metabolites with antibiotic activity might act as microbial defense molecules in natural microbial communities, and that they might also act as cues or signals to trigger gene expression and phenotypic changes at sub-inhibitory concentrations. In applying high-throughput screening on small molecular libraries to find potential inducers for activating silent gene clusters, Seyedsayamdost et al. proved the obvious indigenous activation of two silent gene (*Burkholderia thailandensis*) clusters: the malleilactone cluster and the Burkholdac cluster. They revealed that most initiators themselves were antibiotics. Antibiotics would kill *B. thailandensis* at high concentrations while acting as an inducer of secondary metabolism at low concentrations [43]. Buijs et al. observed an expression of orphan biosynthetic gene clusters in *B. thailandensis* when treated with sub-MIC andrimid which was produced by a marine bacterium *Vibrio corallilyticus*. They tested the effect of sub-inhibitory concentrations of andrimid on another marine Vibrionaceae (*Photobacterium galatheae*). The results showed that andrimid could stimulate the reporter strain *P. galatheae* in a sub-MIC range, and that by UV–visible spectroscopy, andrimid at low concentrations could stimulate the increase in holomycin, a secondary metabolite of *P. galatheae*. Furthermore, in the extract of andrimid-treated *P. galatheae*, they observed a 10-fold up-regulation of an orphan biosynthetic gene cluster, which subsequently resulted in a 1.6–2.2 fold up-regulation of the biosynthesis of holomycin [14].

### 2.4. Pairing with Phototrophs

The basic concept of ecology emphasizes nutrient cycling between phototrophic and heterotrophic organisms. Heterotrophic organisms usually promote the growth of phototrophic organisms in co-culture. This relationship is helpful to establish an effective microalgae–bacteria co-culture model [44,45]. Chhun and his collaborators found that when phototrophs and marine actinobacteria were cultured together as partners for the first time, the photosynthate (released by photosynthetic primary producers) could activate biosynthetic gene clusters of marine actinobacterium *Salinispora tropica*, including the orphan biosynthetic gene clusters *pks3* and *nrps1* [46].

### 2.5. Pairing with Mycolic Acid-Containing Bacteria

Bacteria containing mycolic acid were sometimes selected as paired strains because they could induce pigment production [47]. When the *pks13* gene related to the biosynthesis of mycolic acid in *Tsukamurella pulmonis* TP-B0596 was destroyed, the pigments induced by co-culture of *T. pulmonis* TP-B0596 with *Streptomyces lividans* TK23 were not observed [48]. The co-culture analysis of marine-derived *Aspergillus niger* and a mycolic acid-containing bacteria *Mycobacterium smegmatis* suggested that a pigment produced by *A. niger* was induced, and the cytotoxicity of crude extract on human prostate cancer DU145 cells was significantly increased compared with the mono-culture [49].

### 2.6. Pairing with Microorganisms Containing Halogen Peroxidase-Related Gene Cluster

The high proportion of natural products containing bromine and a small number of chlorine in marine-derived natural products is a prominent characteristic of marine natural products [50]. Halides in seawater are relatively abundant, and marine organisms can usually incorporate these elements into their metabolites through the catalysis of enzymes. However, the statistical results of secondary metabolites of marine-derived microorganisms were interesting. Compared with marine invertebrate natural products (10.1% containing bromine) and marine plant natural products (36.8% containing bromine), only 1.8% of marine microbial natural products contained bromine, and this value is only slightly higher than that (0.7%) which exists in terrestrial microorganisms. Voser et al. proposed that it might be attributed to the presence of a co-culture-like ecosystem around marine-derived microorganisms and microorganisms containing halogen peroxidase-related gene clusters, which promote the halogenation of metabolites [50].

## 3. How to Select the Co-Culture Conditions?

### 3.1. Culture Parameters

#### 3.1.1. Scale of Fermentation

Large-scale co-culture can be carried out on solid agar plates. However, this cultural method has some limitations when a large number of metabolites is needed for structural identification and bioassay. Liquid substrate can also be selected as the medium for the co-culture of strains, commonly known as mixed fermentation [51]. The microscale co-culture method can promote the diversity of strain combinations, facilitate the evaluation of the repeatability, determine the scalability of the culture method, and provide a reference for large-scale fermentation. A total of 65 Micromonosporaceae and bacteria containing mycolic acid were co-cultured or cultured alone by microscale fermentation (500 μL). As a result, 12 Micromonosporaceae across three genera could produce unique metabolites during the co-culture [52].

#### 3.1.2. Optimization of Culture Parameters

Changes in the yield and biological activity of secondary metabolites were observed in the co-culture of autoclaved strains at different growing stages. Considering that both *Aspergillus. terreus* C23-3 and *Aspergillus. unguis* DLEP2008001 can produce effective antibiotics and other bioactive compounds, Wang et al. co-cultured the two strains continuously or simultaneously in a live or inactivation form, respectively, to detect the difference of metabolites. For example, later inoculated *A. terreus* when co-cultured with autoclaved *A. unguis* resulted in a diversity of metabolites, whereas pre-autoclaving *A. terreus* effectively inhibited the growth and metabolism in *A. unguis*. Moreover, the metabolites of *A. terreus* still showed high stability after autoclaving. Their further study demonstrated that co-culture metabolites were usually dominant when the strains were pre-inoculated, and the yields could even be higher than those of mono-culture. Pre-inoculated strains strongly inhibit the growth and metabolism of subsequent inoculated co-culture strains by up-regulating or producing specific secondary metabolites [53]. Jomori et al. compared the induction effects of autoclaved and living *M*. *smegmatis* on marine-derived *A. niger*, and the results showed that the extracts of autoclaved *M. smegmatis* co-cultured with *A. niger* were not significantly different from those of the mono-culture [49]. In 2020, Mokkala et al. co-cultured live or autoclaved *B. subtilis* with five marine-derived fungi (*Eurotium chevalieri*, *Emericella foveolate*, *Myrothecium verrucaria*, *Talaromyces tratensis*, and *Talaromyces stipitatus*), and compared the inhibitory effects of extracts on plant pathogens under different culture conditions. The results showed that co-culture with autoclaved *B. subtilis* significantly reduced the antifungal activity of tested marine-derived fungi [54]. During the co-culture, the growth rate and maximum cell density of the two strains *P. galatheae* and *V. coralliilyticus* were very sensitive to the inoculation rate, and different inoculation rates induced different degrees in stimulating the metabolites of *P. galatheae* and *V. coralliilyticus* [55]. Boruta et al. characterized the secondary metabolites and studied the biological process kinetics of *Aspergillus terreus* ATCC and *Streptomyces noursei* ATCC 11455. They compared the biosynthesis ability of the two strains in co-culture and in mono-cultures. In addition, they established another extensive data set, about the consumption of dissolved oxygen and the uptake rates of glucose and lactose. Generally, it is not surprising to obtain a co-culture model that showed almost no differences in product formation, substrate consumption, and morphological characteristics from the corresponding mono-culture. One of the key issues to be considered when planning co-culture experiments is the dominance of the more “aggressive” strains over their partners. The more “aggressive” strain’s dominance on its partner makes it win in the co-culture. It is essential to design a specific inoculation scheme, namely “Draw”, to prevent the overgrowth of the producing strain. In co-culture, the unexpected “Draw” between *A. terreus* and *S. noursei* was surprising. Compared with other co-culture groups, “Draw” was beneficial for the inoculation plan in stimulating the production of secondary metabolites. The inoculation scheme and medium composition were considered as essential aspects to affect the synergistic development and biosynthetic activity of the two strains. In addition, Boruta et al. designed a methodological approach to evaluate the growth status of co-cultured members by using substrates selectively metabolized in the individual species. In the microbial co-culture system of *S. noursei* and *A. terreus*, since *S. noursei* was proven unable to use lactose, the change level in lactose could reflect the growth of *A. terreus* [56]. In the early stages of co-culture development, lack of repeatability is a factor hindering its development. However, various studies have shown that co-culture can provide repeatable metabolite patterns when the relevant fermentation parameters are firstly optimized and carefully maintained. Wakefield and his collaborators conducted small-scale cultures of bacteria and fungi individually, as well as their co-cultures under different conditions. The fermentation parameters were optimized by liquid chromatography–high resolution electrospray ionization mass spectroscopy (LC-HRESIMS), liquid chromatography-ultraviolet (LC-UV), and microanalysis to maintain the reproducibility of metabolite production. Once the optimized fermentation parameters were determined, large-scale co-culture was conducted to isolate the natural products. The observed small-scale experiments were highly comparable to large-scale fermentations [57]. Rateb et al. isolated 10 secondary metabolites from the co-culture extract of *Aspergillus fumigatus* MBC-F1-10 and *Streptomyces bullii*. In order to understand the possible mechanism of *Streptomyces* inducing the biosynthesis of these fungal metabolites, the effects of medium composition, bacterial biomass extracted by MeOH, bacterial culture broth inactivated by autoclaving, and metabolism of the fungus MBC-F1-10 were studied [58]. Five algicidal tryptamine derivatives were isolated from the co-culture broth of marine *Streptomyces* and *Bacillus mycoides*. In order to solve the low production rates and low reproducibility of metabolites in co-culture, factors such as nutrient composition, culture mode, and pH value of the medium were optimized. The methods of reducing the growth rate gap and slowing down the growth speed were used to prolong the co-culture time of two microorganisms, resulting in an increase in the yield of tryptamine derivatives [59]. Different co-culture methods have been tried to elevate the induction rate of diketopiperazines, including the inoculation times, amounts of two microbes, and the water content of rice medium in the co-culture of the fungus *Penicillium* sp. DT-F29 and the bacteria *Bacillus* sp. B31 [60].

### 3.2. Culture Apparatus

A porous filter was used to physically separate *S. tropica* and *Synechococcus* strains during the co-culture, preventing direct intercellular interactions while allowing the diffusion of small molecules [46]. In the co-culture of *Penicillium* sp. LXY140-R and *Penicillium* sp. LXY140-3, Li et al. designed a dialysis bag separation device, allowing only small molecules to penetrate. Co-culture studies showed that the LXY140-3 strain accumulated trichothecene sesquiterpenes by setting up a co-culture device [61]. For the co-cultivation of the strain, *Streptomyces* sp. MA37 and the Gram-negative bacterium *Pseudomonas* sp., Maglangit and others developed a device consisting of two glass chambers connected by a holding steel clamp and a silicone O-ring. The two culture containers were separated with a polyvinylidene fluoride (PVDF) membrane filter, which permitted the two strains to grow under the same culture conditions but without direct contact. High-performance liquid chromatography (HPLC) traces showed that several unidentified metabolites were up-regulated or expressed in the extracts of co-cultured *P.* sp. and *S*. sp., compared with mono-culture [51].

**Figure 3:**
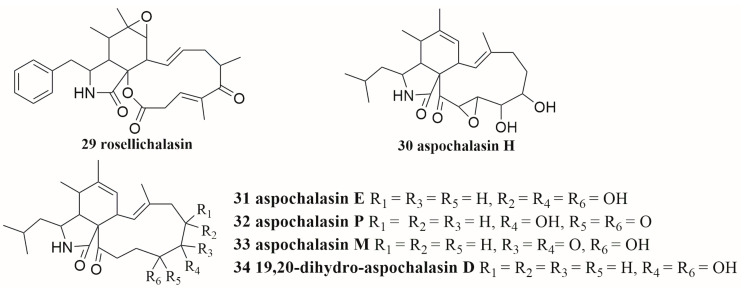
The structures of compounds **29**–**34**.

In some cases, the induction of secondary metabolites requires physical contact between paired strains. The co-cultures of *Aspergillus flavipes* and *Streptomyces* sp. resulted in the up-regulation of cytochalasans (**29**–**34**) (Figure 3), a series of secondary metabolites of *A. flavipes.* In a membrane-separated culture experiment, scanning electron microscopy morphological analysis showed that the effective induction of secondary metabolites required physical contact of microorganisms [62]. In an in-depth study of the co-culture extract of marine-derived *Cosmospora* sp. and phytopathogen *M. oryzae*, Ernest et al. observed the induced isochromanones and their accumulation in the antagonistic zone in the overlaid co-culture on solid agar, suggesting that direct intercellular contact between *C.* sp and *M. oryzae* may be critical for isochromanones’ production on PDA media [37]. The co-cultivation of *A. niger* and *M. smegmatis* led to the production of a pigment by *A. niger*. The extract of co-culture exhibited increased cytotoxic activity against human prostate cancer DU145 cells. Pigment would be produced by *A. niger* and not be observed in an *A. niger* and *M. smegmatis* combined culture mode treated in two separated compartments partitioned with a dialysis membrane. The cytotoxicity of the extract was not significantly different from that in axenic culture of *A. niger*. Similarly, the extracts of autoclaved *M. smegmatis* co-cultured with *A. niger* were not significantly different from those of the mono-culture. Therefore, it was necessary to co-culture living *M. smegmatis* and *A. niger* in cell–cell interaction [49].

## 4. How to Screen the Chemical Diversity of Co-Culture Extracts?

### 4.1. Morphological Interactions

**Figure 4:**
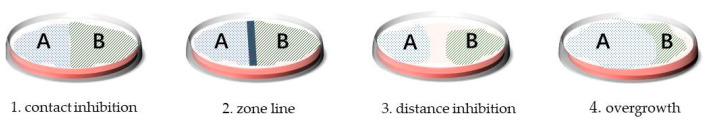
Morphological interactions among two fungi. The two co-cultured fungi were represented by “A” and “B”, respectively. The blue region refers to the growth region of strain A, and the green region refers to the growth region of strain B.

Generally, fungi adapt to growing on solid media by extending their hyphae into undeveloped regions containing nutrients. Morphologically, the interaction between the two fungi in a co-culture experiment on solid agar plates can be observed. Four types of interaction were observed on the agar plates in the co-cultures of phytopathogens and the selected marine-adapted fungi. These interaction types were also reported by Bertrand et al. in 2014 [16] and Ernest et al. in 2020 [38] (Figure 4): gaps are observed between strains (distance inhibition), distinct lines are observed between strains (zone line), the strains are in contact with each other (contact inhibition), and one strain is overgrown with the other (overgrowth) [16,36,38]. The ultra-high-performance liquid chromatography-quadrupole time-of-flight tandem mass spectrometry (UPLC-QTOF-MS/MS) analysis of the chemical constituents in the confrontation zone of the co-culture of marine-adapted fungus *Cosmospora* sp. and phytopathogenic fungus *M. oryzae*. confirmed that there were three compounds producing via co-culture and they were overexpressed in the confrontation zone and one novel compound was present in the confrontation zone, highlighting the importance of co-culture [36].

### 4.2. TLC and HPLC Fingerprints

The analysis of HPLC or thin-layer chromatography (TLC) fingerprints reveals significant variation between co-cultured and mono-cultured extracts [63]. By comparing the HPLC chromatograms of the metabolites of *Streptomyces.* sp. and *A. flavipes* through mono-culture and co-culture, six induced compounds (**29**–**34**, Figure 3) were isolated and identified only in co-culture [62]. 

### 4.3. MS and Other Methods

Liquid chromatograph mass spectrometer (LC-MS) is a more sensitive analytical method than HPLC fingerprinting. It can detect compounds with low response values [60]. Several trace metabolites have been detected using LC-HRMS metabolomics analysis in co-culture extracts from two sponge-derived actinobacteria: *Actinokineospora spheciospongiae* strain EG49 and *Rhodococcus* sp. UR59 [64].

As a general approach, LC-MS combined with principal component analysis (PCA) was used to compare the differences in crude extracts of co-cultured and mono-culture strains. Pareto scaling provides a trade-off between the unmodified data and the dimensionless unit variance scaling, reducing the importance of high-intensity compounds and helping to identify low-intensity ions. It serves as a reference scale for evaluating secondary metabolite changes in PCA [52].

Based on UPLC-HRMS/MS data, global molecular networks generated by the Global Natural Products Social Molecular Networking platform can capture different chemical structure classes and similar compounds into different clusters, regardless of retention time, type of extraction, or medium. This approach allows for the rapid identification of putative new compounds and new derivatives of known compounds [61]. Twenty-one fungal strains with genetic diversity were selected to co-culture with two phytopathogenic bacteria (*P. syringae* and *R. solanacearum*) and two phytopathogenic fungi (*M. oryzae* and *B. cinerea*) in two solid media (SA and PDA). Twenty-one fungi were isolated from the samples of water, sediment, seabed foam, and plant materials (seeds, branches, leaves, and sawdust) collected at the Windebyer Noor. Based on the global molecular network generated by UPLC-HRMS/MS data, supplemented by the freely available universal natural products database (ISDB-UNPD) and manual dereplication, all co-cultured and mono-cultured extracts of corresponding marine-adapted fungi and plant pathogens were deduplicated. The co-culture of marine-adapted fungus *Emericellopsis* sp. and the phytopathogen *M. oryzaegave* gave the largest cluster in the global molecular networks, and the induced peaks responsible for the variation between the co-cultures and mono-cultures were visualized by an S-plot combining covariance and correlation. A quarter of the data in the S-plot’s upper right corner represent ions induced in co-culture. In contrast, a quarter of the labeled ions in the lower left corner represent unique ones in mono-culture. The ions identified only in co-culture indicated the formation of new compounds. The repeatability of the experiment was ensured by measuring the three biological replicates of each culture, and the results were visualized by the principal component analysis scores’ plot [36]. In some cases, the yield of some compounds identified by the molecular network is so low that no other relevant structural identification information about these compounds can be subsequently determined. In addition, compounds with low proton fragmentation density cannot be successfully visualized in a molecular network.

Matrix-assisted laser desorption/ionization imaging mass spectrometry (MALDI-IMS) is a powerful technique that can simultaneously visualize the spatial and temporal distribution of multiple metabolites produced directly by microorganisms on agar rather than be focusing on individual molecules or pathways. MALDI-IMS, in combination with a molecular network, has been used to identify key metabolic exchange factors in the interactions of two co-cultured strains, and to reveal the role of these metabolites in the regulation of polymicrobial systems [65].

## 5. Activation of Biosynthetic Gene Clusters in Co-Cultured Strains

Chhun and his collaborators found that the photosynthate released by photosynthetic primary producers could activate biosynthetic gene clusters of marine actinobacterium *S. tropica*, including the orphan BGCs *pks3* and *nrps1*. Furthermore, they provided evidence that *S.tropica*’s *nrps1* may produce a promising antimicrobial compound acting as a ClpP proteasome inhibitor. Unfortunately, the structure of the cryptic metabolites had not been elucidated. The instability and the lower yields of this compound were the possible reasons that hindered its isolation [46].

Buijs et al. treated marine-derived strain *P. galatheae* (in sub-MIC) with andrimid, the acetyl-CoA carboxylase inhibitor produced by marine bacterium *V. coralliilyticus*. A 10-fold up-regulation of an orphan BGC and a 1.6–2.2-fold up-regulation of the gene that encodes the core enzyme for holomycin biosynthesis were observed in andrimid-treated *P. galatheae* cultures by means of transcription and chemical measurements. In addition, andrimid did indeed induce the production of holomycin by *P. galatheae*. Competition sensing provides some explanation to the ecological mechanism in which antibiotics induced secondary metabolite production. The theory suggests that bacteria can detect competitors through their dangerous natural products and respond with favorable coping strategies such as increasing their own antibiotic production or forming defense biofilm. Andrimid-treated *P. galatheae* cultures induced a general (*rpoS*) stress response at the transcriptional level and increased the overproduction of holomycin [14,66]. Subsequently, they investigated the secondary metabolites produced by *V. coralliilyticus* and *P. galatheae* under co-culture conditions. In the co-culture, the amounts of andrimid (product of *V. coralliilyticus*) and holomycin (product of *P. galatheae*) increased 4.3 and 2.7 fold, respectively, as compared with the mono-cultural products. Moreover, dimethyl-holomycin, a methyl-detoxification strategy to protect *V. coralliilyticus* from the influence of holomycin, was observed in co-cultures [55].

Wang et al. co-cultured two strains, *A. terreus* and *A. unguis*, continuously or simultaneously in live or inactivation form, respectively. These strains significantly down-regulated several metabolites that had no or only weak antimicrobial activity, presumably via saving unused metabolic pathways under stress conditions. Only when inoculated simultaneously with or later than *A. terreus*, *A. unguis* will produce new dichlorinated metabolites, which may be related to the chemical defense capability of these chlorinated natural products in this fungus [53]. UPLC-MS analysis of the co-culture extracts of *P. influorescens* and *P. nobilis* showed unexpected and significantly suppressed peaks and a newly induced peak compared to mono-culture throughout the entire co-culture process. These observations were highly suggestive for a competitive interaction involving the suppression of biosynthetic pathways of one strain, and up-regulation of highly functional metabolites which mediate the competitive interaction by the other [38]. In the co-culture system of *Aspergillus sclerotiorum* DX9 and *Streptomyces* sp. WU20, the presence of cyclo(Pro-Trp) produced by *Streptomyces.* sp. may lead to the increase in notoamides (produced by *A. sclerotiorum*). Further amino acid feeding experiments suggested that *A. sclerotiorum* could utilize the cyclo(Pro-Trp) and convert the latter into a synthetic precursor of notoamides [67].

## 6. Chemical and Activity Diversity of Secondary Metabolites

**Figure 5:**
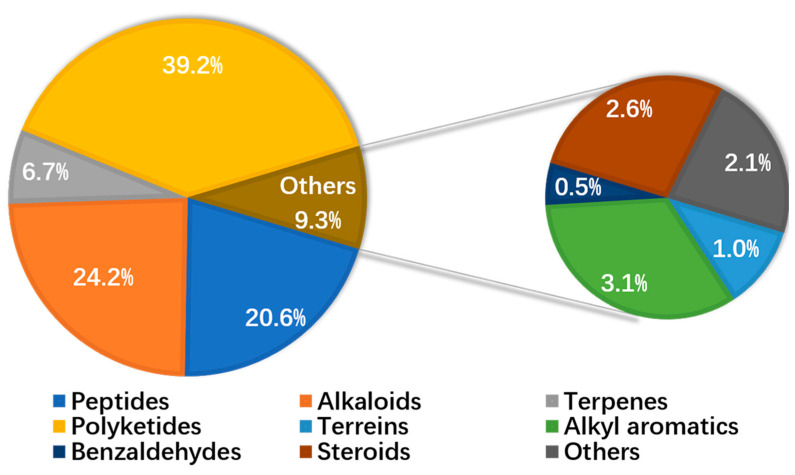
Distribution of the compounds according to chemical structure.

This review concluded a total of 194 compounds with a wide range of activities of cytotoxicity, antibacterial, antifungal, antimalarial, anti-fouling, and antioxidation, etc. Secondary metabolites isolated from marine-derived co-culture microorganisms are dominated by polyketides (39.2%), alkaloids (24.2%), and peptides (20.6%) (Figure 5).

### 6.1. Peptides

**Figure 6:**
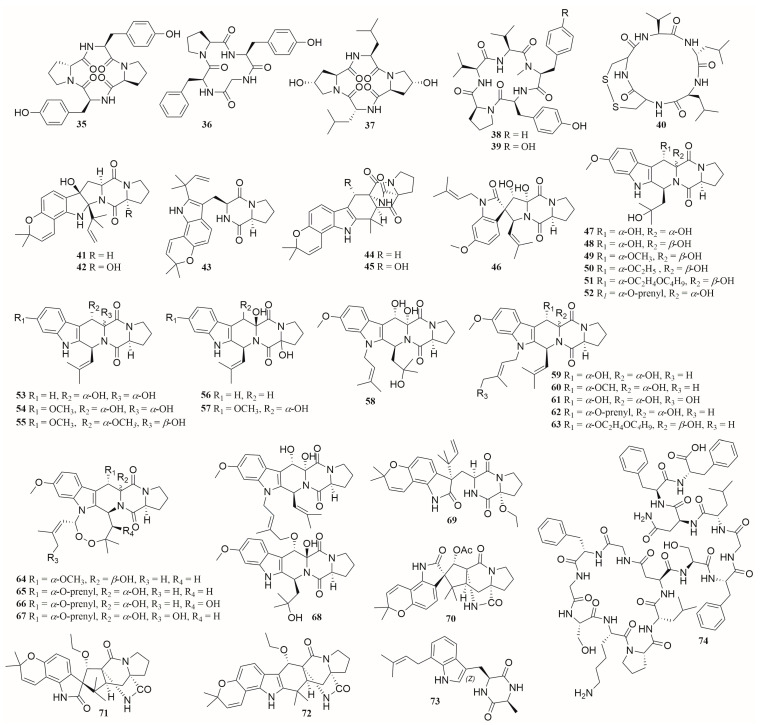
The structures of compounds **35**–**74**.

**Figure 7:**
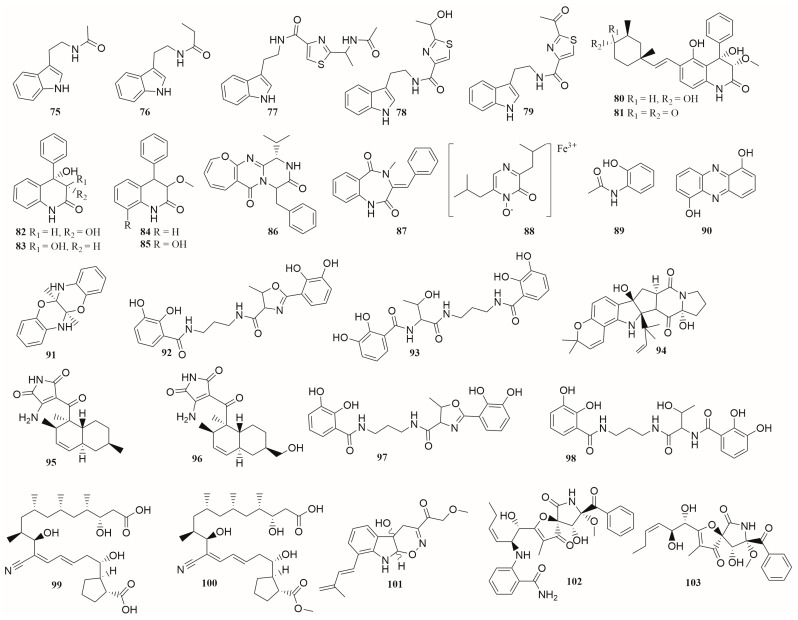
The structures of compounds **75**–**103**.

From 2012 to 2022, a total of 33 cyclic dipeptides (**41**–**73** in Figure 6) and 7 cyclic peptides (**35**–**40**, **74** in Figure 6), including 17 new compounds (**35**, **38**, **50**, **51**, **56**, **57**, **61**, **63**, **64**–**72**), were isolated from co-cultured extracts of different marine-derived strains. These co-cultured microorganisms include fungi from four genera, *Phomopsis*, *Alternaria*, *Aspergillus,* and *Penicillium*, and bacteria from three genera, *Bacillus*, *Mycobacterium*, and *Streptomyces*. These peptides exhibit diverse biological activities such as growth inhibition of fungal or bacterial, antioxidation, and cytotoxicity [49,57,60,68,69,70,71,72]. Compound **40** showed strong cytotoxic activity by blocking the growth of cancer cells colon 38 and HCT 116 in G2/M phase, with the IC_50_ values of 0.27 ± 0.07 μM and 0.18 ± 0.023 μM, respectively [49,73].

### 6.2. Alkaloids

In exploring the diversity of secondary metabolites of marine-derived strains based on co-culture patterns, a total of 47 alkaloids and other nitrogen-containing compounds (**1**–**6**, **9**–**12**, **14**, and **15** in Figure 1; **29**–**34** in Figure 3; **75**–**103** in Figure 7) were isolated and identified, including 15 new compounds (**3**–**6**, **9**–**11**, **14**, **15**, **80**, **94**, **99**–**102**). These co-cultured microorganisms producing alkaloids include fungi from three genera, including *Aspergillus*, *Isaria,* and *Penicillium*, and bacteria from ten genera, including *Streptomyces*, *Bacillus, Saccharomonospora*, *Dietzia*, *Actinokineospora*, *Nocardiopsis*, *Achromobacter*, *Serratia*, *Shewanella*, and *Rhinocladiella* [29,30,31,57,59,62,70,71,72,74,75].

These alkaloids exhibit diverse biological activities such as growth inhibition of fungi or bacteria, kinase inhibitory activity, or cytotoxicity. Compound **5** showed cytotoxicity against the human cell line U937 and toxicity against brine shrimp with the IC_50_ values of 4.2 μM and 6.1 μM, respectively [29]. In the antibacterial activity test, compound **99** showed a strong inhibitory activity against MRSA with an MIC value of 0.195 μg·mL^−1^ [72].

As a nutrient, iron is essential for biological processes such as respiration, gene regulation, and DNA biosynthesis. In iron-limited environments, microorganisms produce large quantities of iron-chelating compounds called siderophores. A high yield of compound **92** was observed in the iron-limited co-culture extract of two bacteria, *Shewanella* sp. and *Serratia* sp., which was not found in the iron-added co-culture of two of these bacteria, nor in the axenic cultures of the bacteria [75].

### 6.3. Terpenes

**Figure 8:**
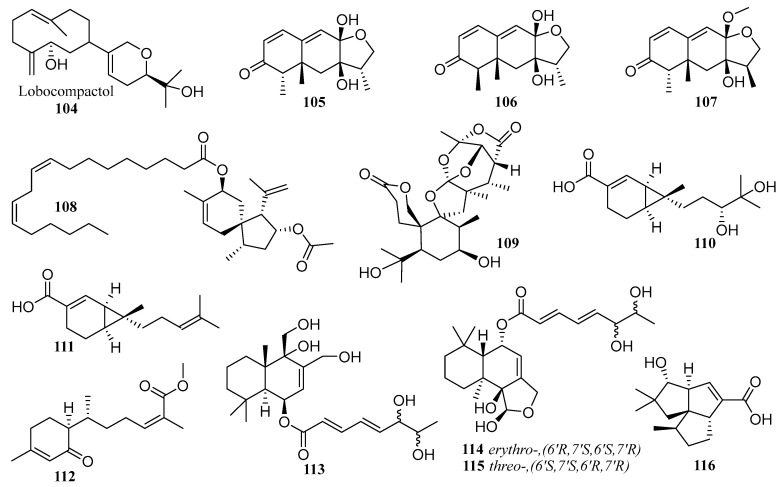
The structures of compounds **104**–**116**.

Thirteen terpenoids (**104**–**116**), including nine novel compounds (**104**–**106**, **108**, **109**, **113**–**116**) (Figure 8), were isolated from co-cultured fermented extracts of different marine strains [57,61,76,77,78,79]. These co-cultured microorganisms producing terpenoids included fungi from four genera, including *Penicillium*, *Trichoderma, Aspergillus,* and *Beauveria,* and bacteria from three genera, including *Streptomyces*, *Alteromonas*, and *Acinetobacter*. These terpenoids enriched the activity information of natural products, such as anti-fouling, inhibiting α-glycosidase, and inhibiting bacterial and fungal growth. Considering the activity against primary fouling organisms such as macroalgae zoospores, diatoms, and bacteria, compound **104** showed inhibition to the sedimentation of *Ulva pertusa* spores and sensitivities to bacteria KNP-5 and KNP-8, suggesting that it might be useful in the antifouling industry [76]. The meroterpenoid derivative **108**, isolated from the co-cultured extract of *Penicillium bilaiae* MA-267 and *Penicillium chermesinum* EN-480, showed strong inhibitory activity against *Edwardsiella tarda* and *Ceratobasidium cornigerum* with MIC values of 0.25 and 0.5 μg·mL^−1^, reactively [78].

### 6.4. Polyketides

**Figure 9:**
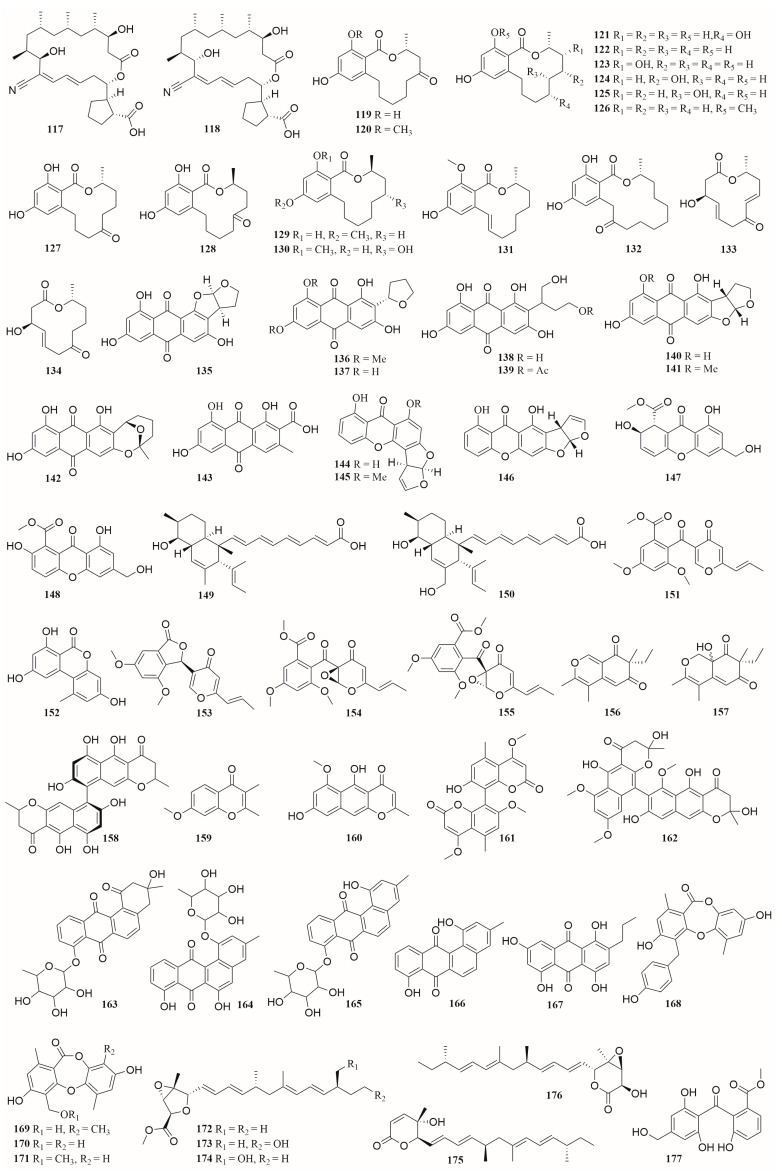
The structures of compounds **117**–**177**.

A total of 76 polyketides (**7**, **8**, **13** in Figure 1, **16**–**24**, **26**–**28** in Figure 1, **117**–**177** in Figure 9), including 19 macrolides (**13**, **117**–**134**) and 57 other polyketides (**7**, **8**, **16**–**24**, **26**–**28**, **135**–**177**), were produced by the co-culture of microorganisms isolated from marine environment. Among them, 17 compounds were new (**7**, **8**, **19**, **20**, **119**, **121**, **134**, **135**, **136**, **149**, **150**, **157**, **168**, **172**, **173**–**175**). These microorganisms were grouped into 9 genera of fungi, including *Rhinocladiella*, *Trichoderma*, *Aspergillus, Penicillium, Plenodomus, Pyrenochaeta, Cosmospora*, *Magnaporthe*, and *Talaromyces*, and 10 genera of bacteria, including *Streptomyces*, *Acinetobacter*, *Saccharomonospora*, *Dietzia, Bacillus, Janthinobacterium, Mycobacterium, Staphylococcus*, *Pseudomonas*, and *Rhodococcus* [29,30,31,37,38,40,41,49,64,70,72,77,80,81,82,83].

These polyketides exhibit a variety of biological activities such as α-glucosidase inhibition, cytotoxicity, antimalarial, anti-plant pathogens, and antibacterial. Compounds **121** and **119** showed strong inhibitory activity against α-glucosidase with IC_50_ values of 25.8 and 54.6 μM, respectively [77]. Compound **155** showed cytotoxic activity towards HL-60 and H1975 tumor cells with IC_50_ values of 3.73 and 5.73 µM, respectively. Compound **154** showed cytotoxic activity towards H1975 cells with IC_50_ value of 3.97 µM [81]. Compound **158** inhibited the growth of *X. campestris* and *P. infestans* with IC_50_ values of 0.9 and 1.7 μg·mL^−1^, respectively [38]. Compound **21** exhibited antifungal activity against *P. syringae*, *X. campestris*, *M. oryzae,* and *P. infestans* with IC_50_ values from 0.8 to 23.4 µg·mL^−1^ [37]. Compounds **163**–**167** showed antimalarial activity, with IC_50_ values ranging from 9.0 to 13.5 μg·mL^−1^ [64]. Compounds **168**–**170** exhibited α-glucosidase inhibitory activity with IC_50_ ranging from 8.1 to 11.2 μM. Compound **168** displayed cytotoxicity against MMQ and GH3 cell lines with IC_50_ values of 3.09 and 3.64 μM, respectively [82]. Compound **175** showed cytotoxic activity against human multiple cancer cell lines including HeLa, K562, HCT-116, HL-60, A549, and MCF-7 with IC_50_ values of 5.5, 2.9, 1.4, 1.2, 5.1, and 9.8 μM, respectively [83].

### 6.5. Others

**Figure 10:**
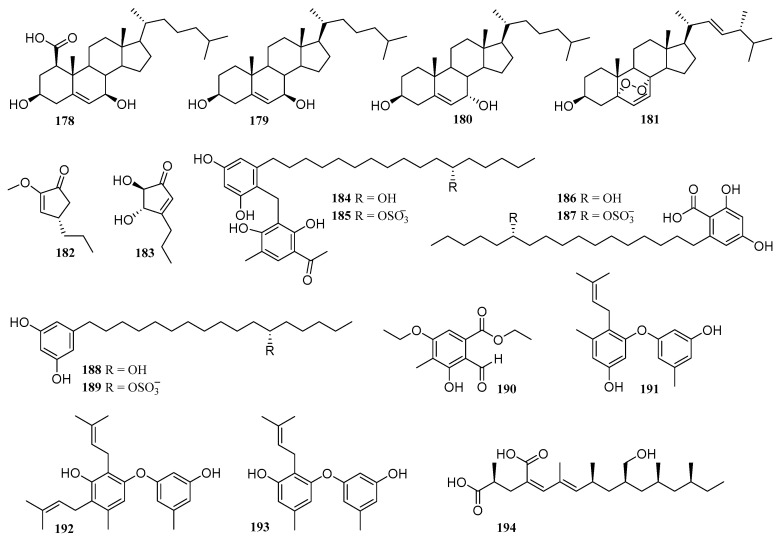
The structures of compounds **178**–**194**.

In addition to the above three types of compounds, 18 compounds were isolated and identified from co-cultured fermented extracts of marine-derived strains (**25** in Figure 2, **178**–**194** in Figure 10), including terrein derivatives, alkyl aromatics compounds, benzaldehyde derivatives, and steroids, of which 7 were new compounds (**178**, **182**, **184**–**187**, **190**) [37,70,84,85,86,87,88]. These compounds exhibited a variety of biological activities, such as cytotoxicity, antibacterial, and antifungal activity. The above co-cultured microorganisms include fungi from six genera, including *Aspergillus*, *Paecilomyces, Penicillium*, *Xylaria*, *Cosmospora*, and *Magnaporthe,* and bacteria from two genera, including *Leeuwenhoekii* and *Bacillus*.

## 7. Challenges and Prospects

Marine ecosystems are characterized by the complexity interactions between two or more types of microorganisms that form an intricate, dynamic ecological network through the continuous exchange of material, energy, and information between cells [89], and thus challenges arise in the construction of marine-derived microbial co-culture models, as well as in tracing the process and results of co-culture. These challenges mainly include full understanding of marine-derived microbial ecological networks and the roles of the paired strains and the main or significant bioactive metabolites in the co-culture system. During fermentation, there are also problems in adjusting the heterogeneous growth rates of strains, balancing the different nutritional requirements, reducing the overgrowth of dominant strains, and realizing the repeatability and scale up [90]. No complete co-culture model has yet to be established, and most of the co-culture induction strategies are still based on serendipity [90]. Several biosynthetic gene clusters have been successfully activated in a few co-culture cases, but the structural characterization of their induced secondary metabolites is often unclear due to low yields. At present, the fermentation of secondary metabolites in the co-culture system is only carried out on relatively small scales such as shake flasks and plates.

Some promising methods may be helpful for the analysis of the interaction of paired strains. High-throughput biotechnologies such as genome, transcriptome, metabolome, and proteome have been used to study the mechanism of polyketides produced in microbial co-culture system. In addition, genome-scale metabolic models have been used to predict the metabolic flux of the co-culture system, which is helpful for manual intervention in the co-culture system [91]. The investigation of interactions between complex marine-derived microbial species based on synthetic biology and ecology may provide some references for constructing co-culture models, analyzing strain growth properties, and studying biosynthetic pathways for secondary metabolites. Moreover, some emerging culture techniques, such as membrane diffusion-based culture methods, can mimic the natural environments as much as possible during co-culture [91]. The state-of-the-art AI technology will also play an important role in the near future. Due to the problem of a few novel bioactive molecules (accompanied by a large amount of known ones) which have been discovered by using mono-cultures, and with the development of molecular biology, genetics, computer science, gene-editing technology, and fine precise devices (for fermentation, detection, or analysis), co-culture strategies by using two or more types of marine microorganisms are promising and will play vital roles in exploring novel bioactive secondary metabolites. As more marine-derived microorganisms are isolated and identified in the ongoing ocean exploration, we expect that more structurally novel secondary metabolites with fruitful bioactivity will be discovered soon.

## 8. Conclusions

Marine-derived microbial natural resources are becoming an important field in searching for structurally novel and bioactive drug molecules [92]. In the past years, an astonishing number of new structures have been discovered [89,92,93,94,95]. Considering the particular marine environment, many scientists have tried to apply the co-culture strategy to marine-derived microorganisms for the discovery of novel bioactive compounds. This paper focuses on the co-culture of marine-derived microorganisms in the last 10 years. The selection of paired strains, co-culture parameters, detection methods of secondary metabolites, and the structural diversity and activity of the metabolites have been reviewed. Due to the unavoidable shortcomings of mono-culture, co-culture strategy has significant privileges in the discovery of novel bioactive metabolites; however, tremendous work is needed to be carried out before stable, system, and mature co-culture models are established. Once the co-culture strategy is successful, it will bring us druggable molecules that are beneficial for the treatment of human various diseases.

## Data Availability

The original data presented in the study are included in the article; further inquiries can be directed to the corresponding author.

## Abbreviations

UV	Ultraviolet
OSMAC	One strain-many compound
SA	Sabouraud medium
PDA	Potato dextrose agar medium
MIC	Minimal inhibitory concentration
MRSA	Methicillin-resistant Staphylococcus aureus
HRESIMS	High resolution electrospray ionization mass spectroscopy
LC	Liquid chromatography
PVDF	Polyvinylidene fluoride
HPLC	High-performance liquid chromatography
UPLC	Ultra-high-performance liquid chromatography
QTOF	Quadrupole time of flight
MS/MS	Tandem mass spectrometry
TLC	Thin-layer chromatography
MS	Mass spectrometer
PCA	Principal component analysis
MALDI-IMS	Matrix-assisted laser desorption/ionization imaging mass spectrometry
